# Review of Tailoring Methods for Joints with Additively Manufactured Adherends and Adhesives

**DOI:** 10.3390/ma13183949

**Published:** 2020-09-07

**Authors:** Mattia Frascio, Eduardo André de Sousa Marques, Ricardo João Camilo Carbas, Lucas Filipe Martins da Silva, Margherita Monti, Massimiliano Avalle

**Affiliations:** 1 Polytechnic School, University of Genoa, 16145 Genoa, Italy; m.monti@unige.it (M.M.); massimiliano.avalle@unige.it (M.A.); 2 Instituto de Ciência e Inovação em Engenharia Mecânica e Engenharia Industrial (INEGI), 4200-465 Porto, Portugal; emarques@fe.up.pt (E.A.d.S.M.); carbas@fe.up.pt (R.J.C.C.); lucas@fe.up.pt (L.F.M.d.S.); 3 Departamento de Engenharia Mecânica, Faculdade de Engenharia (FEUP), Universidade do Porto, 4200-465 Porto, Portugal

**Keywords:** additive manufacturing (AM), adhesively bonded joint, polymer, fused filament fabrication (FFF), selective laser sintering (SLS), polijet, photo-polymerization, design for AM, review

## Abstract

This review aims to assess the current modelling and experimental achievements in the design for additive manufacturing of bonded joints, providing a summary of the current state of the art. To limit its scope, the document is focused only on polymeric additive manufacturing processes. As a result, this review paper contains a structured collection of the tailoring methods adopted for additively manufactured adherends and adhesives with the aim of maximizing bonded joint performance. The intent is, setting the state of the art, to produce an overview useful to identify the new opportunities provided by recent progresses in the design for additive manufacturing, additive manufacturing processes and materials’ developments.

## 1. Introduction

The industrial interest in additive manufacturing (AM) has been a driving factor behind the development of modern 3D printer machines [1] and their novel features, such as multi material AM (MMAM) simultaneous processing [2]. This interest has led to increased process standardization [3] besides the adoption of new design approaches for additive manufacturing (DfAM) [4], and new materials. DfAM urges us to rethink component design by taking in account both the specific constraints and new opportunities created by the AM processes. Many studies have analyzed these constraints, demonstrating how positioning for printability, printing parameters and printing set ups can affect the surface finishing, geometrical accuracy and the mechanical properties of the resultant components [4,5,6,7]. Therefore, the nominally limitless geometrical freedom of AM is in fact restricted by an important set of design parameters.

As the knowledge in DfAM increases, so too does the potential of AM. Although still limited by a relatively modest production rate, if compared to traditional manufacturing processes, AM finds practical use in the production of highly optimized components of limited size to be assembled in larger structures [8]. Moreover, this approach addresses the exponential relationship between building volume and printer cost and allows us to improve productivity using several 3D printers working in parallel, avoiding complete stoppages of production during maintenance [9]. However, the design approach that focuses on splitting the components in order to overcome current AM processes limitations introduced above, requires the use of an effective joining procedure to create a larger final product from the smaller subassemblies.

One of the earliest works on joining AM components was carried out by Espalin et al. [10], in which the authors identified the techniques suitable for assembling polymeric AM components, which include ultrasonic spot welding, hot air welding, solvent joining and adhesive joining, and another was carried out by Arenas et al. [11], in which a method was proposed to select structural adhesives for AM.

Subsequent studies explored joint performance improvements using AM as a joining tool, the AddJoining process [12,13] with or without co-curing, and a voxel-oriented design [14] approach. A hybrid approach combining the first two solutions was also considered. The AddJoining process consists in 3D printing the substrates and the joint simultaneously, using the structure as the build plate. The voxel-oriented design approach consists in exploring the local material control conferred by AM with previously acquired know how on adhesive bonding [15] to develop new joint design solutions.

After the introduction, the existing literature is sorted according to the design strategy used by authors and details are given for each of these techniques. In the conclusion section, the advantages and disadvantages of the proposed techniques are summarized, and some general remarks are drawn.

## 2. Joint Design Strategies for Additive Manufacturing

**Figure 1:**
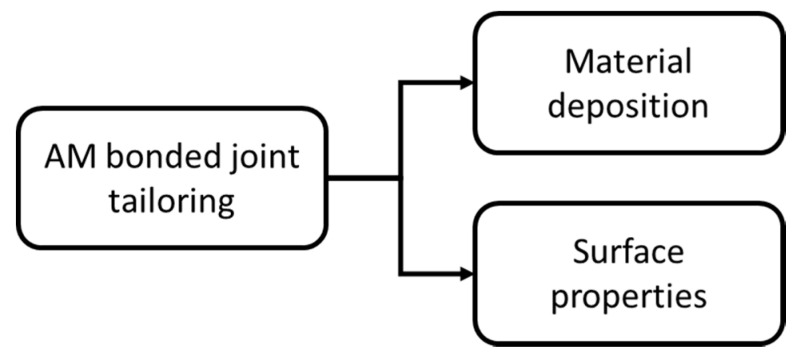
Macro sorting of the design strategies for additive manufacturing (AM) bonded joints.

The process of optimizing the design of bonded joints can be briefly described as maximizing the load carrying capacity of assembled components in order to withstand higher service loads. Initially, joint design strategies were based on selecting materials with better material properties and on maximizing the bonding area. Later, with the growing need for developing lightweight structures, the investigations on the physics of the adhesion and the advances in modelling techniques enabled the development of alternative joint design strategies [16]. These design strategies are based on a better understanding of stresses acting on the adhesive layer, on the adhesive and of the effect of adherends material properties. This knowledge is implemented in novel geometrical configurations in order to lower peel and cleavage stresses [17]. Even if the overall joint design is a compromise between an optimal geometrical configuration and constraints on components sizes, more localized solutions can be implemented in order to change the stress distribution in the bondline. Adhesive stiffness is a major factor that affects the stress distribution along the overlap [18], thus a locally modified adhesive [19] can improve the strain tolerance of the joint and delay failure. Another option to improve the stress distribution in the adhesive can be to locally modify the adherend geometry [20] achieved; for example, by tapering the adherends at the overlap ends to lower the stiffness, and thus stress in these critical locations. Another approach that can be used to improve the level of adhesion [21] is the use of chemical or mechanical treatments, to modify the bonding surfaces. All of these design concepts can be taken to an extreme by exploiting the material properties and geometrical AM controlled at the voxel resolution [14]. In this section, the use of AM to improve joint design is discussed in detail, sorting the works as detailed in Figure 1.

### 2.1. Material Tailoring

The first design strategy discussed is the use of AM to locally change the material properties in the joint. This approach can be implemented by simultaneous deposition of different materials, for example with MMAM [2] in order to obtain functionally graded adherends and adhesives. This is a design concept that has already been investigated for other manufacturing processes [19,22]. Another option can be using AM to create complex structures in the thickness direction, such as a lattice, cellular or auxetic structures, which will guide the deformation under load of the joint and affect the modulus by introducing controlled porosity.

**Figure 2:**
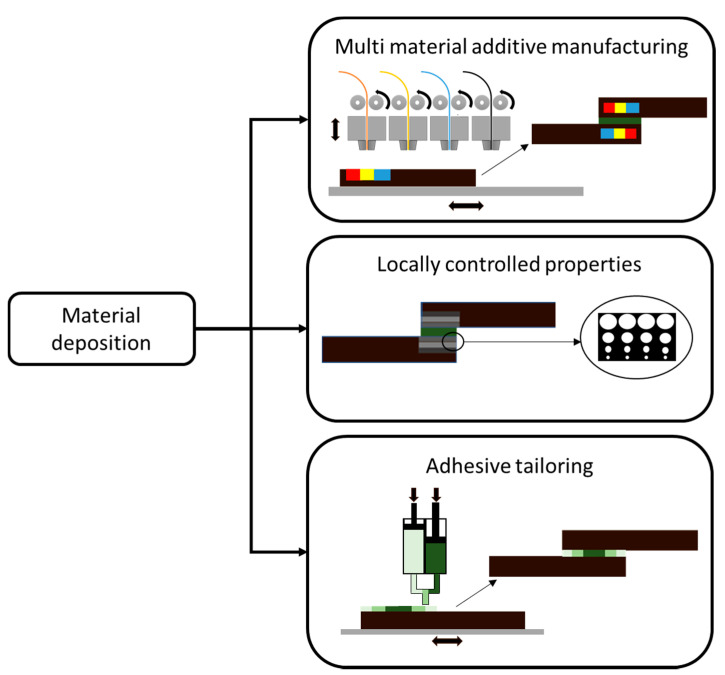
Voxel oriented material deposition design strategies for AM bonded joints.

In the following sub sections, the works concerning AM tailored materials and joints shown in Figure 2 are presented.

#### 2.1.1. Multi Material Additive Manufacturing

Kumar et al. [23,24] and Khan et al. [25,26] used AM to create single lap joint (SLJ) geometries with tailored modulus in the bondline layer without additional joining, a process conceptually similar to that of AddJoining [12].

In a preliminary study, Khan et al. [27] implemented an analytical model to investigate the effect of tailoring the elastic modulus along the full length of SLJs. The aim was to evaluate the effect of introducing the additional design variable of material compliance using the MMAM processes. A general approach applicable to adherend and adhesive has been developed, based on the Euler-Bernoulli and Timoshenko beam theories, to obtain a sandwich structure representative of an SLJ. From a parametric analysis, carried out using the analytical model, the authors were able to show several configurations in which, using MMAM elastic modulus tailoring, stress peaks at the overlap ends were reduced and, therefore, the overall load carrying capacity of the joints was enhanced. In subsequent works, authors used the general analytical model applied to several joint configurations and then experimentally assessed the effect of the proposed stiffness tailoring approach.

**Figure 3:**
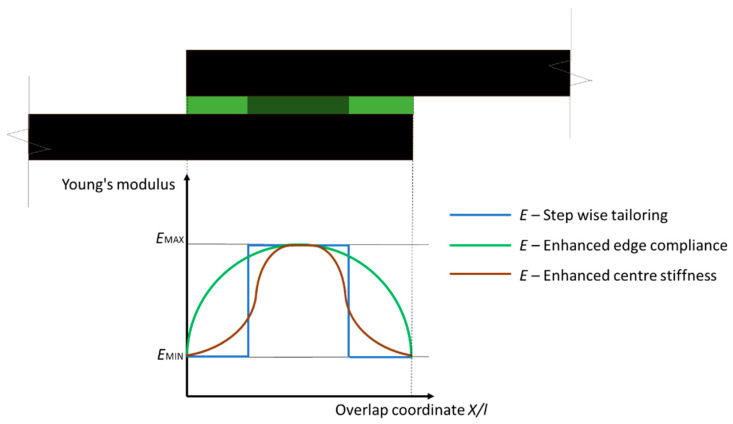
Bond line modulus tailoring using digital materials for single lap joints (SLJs) [23,24].

Kumar et al. [24] first explored the effect of using a material with a lower elastic modulus at the overlap ends with a step wise pattern (Figure 3). The focus was on the optimization of the overlap stress distribution using the volumetric percentage *v_c_* of low-stiffness material of the total material in the bondline as a design parameter. Taking advantage of the photopolymerization process resolution, the authors were able to obtain two digital materials (DMs) with hybrid characteristics in respect to the bulk materials [28], with an elastic modulus ratio between the high-stiffness material and the low-stiffness material of around 2. A first investigation using finite element method (FEM) of the SLJs indicated a beneficial effect of the material tailoring on the shear and peel stresses distributions at the overlap ends, with minimum stress values being achieved for *v_c_* values in between 0.1 and 0.2. However, a detrimental effect at the material interface was also reported, due to a stiffness mismatch. Experimental tests were carried out on SLJs for *v_c_* values between 0 and 1 for a discrete step increase of 0.2. Results confirmed that the best configuration is for *v_c_* equal to 0.2 with joint performance improvement of 23% on ultimate load and 37% on toughness in respect to the solution with only the stiffer material.

In a following work [23], the authors improved the design of the modulus tailoring, seeking a gradual variation along the overlap (Figure 3) mimicking with the DMs the structure of a second phase dispersion in the adhesive. Reference SLJs were fabricated with neat homogeneous material, in order to assess the effect of the modulus tailoring, and with the spheres of the dissimilar material evenly distributed over the overlap zone. Experimental results were similar for both tailored configurations, with a maximum load increment of about 100% and a joint toughness increment of 169% in respect to the standard configuration. The results also demonstrated that modulus tailoring can change the failure mode, from adhesive to cohesive due to the reduction in peel stresses. It is worth noting that the presence of spheres has a beneficial crack arresting effect even if the interface between the different material can be considered as a flaw in the adhesive. Moreover, even if the process resolution in the layer was about 50 µm, authors had to use geometrical features at least 10 times larger than the minimum resolution in order to respect design tolerances. This technical constraint led to the use of thicknesses larger than those usual in SLJ manufacture.

**Figure 4:**
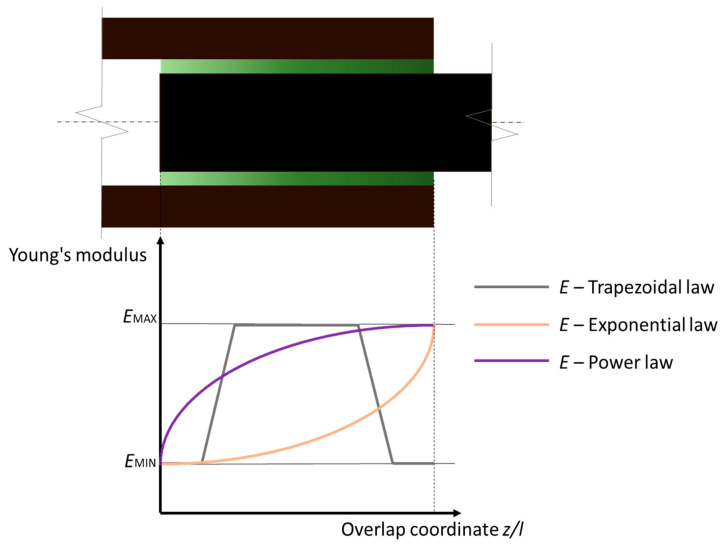
Seamless bond line modulus tailoring using DMs for tube-shaft joint [25,26].

The works of Khan et al. [25,26] focused on the compliance tailoring of joints in cylindrical configurations using three different strategies for the gradation of the elastic modulus of the adhesive layer. These were the use of power law, exponential law and trapezoidal law (Figure 4). The authors developed a dedicated specimen geometry to test shaft tube joints under axial tensile loads with dimensions compatible with the resolution of the photopolymerization process. Tailoring strategies were based on spherical inclusions of DMs. At first, numerical and analytical analyses were carried out in order to investigate the best gradation coefficients for each elastic modulus design law [29]. In [25], authors observed that stress peaks at the tube end are greater than at the shaft ends, thus implemented monotonic elastic modulus variations using a power law and an exponential law. The bond layer elastic modulus at the overlap was tailored as a function of the axial coordinate between is the lowest elastic modulus (*E*_MIN_ = 280 MPa) and the highest elastic modulus (*E*_MAX_ = 2700 MPa). Numerical and analytical models pointed out different optimal configurations for the different gradation laws. Experiments confirmed the model results, showing a 40% increment in the failure load and a 25% increment of toughness in respect to the non-tailored configuration.

In [26], authors investigated the effect of two-sided linearly varying elastic modulus profiles created using a trapezoidal law. The model output gives best results for adhesive gradation normalized on the overlap length equal to 0.25. Experiments on this configuration were compared to baseline solutions with homogeneous bond layers with elastic modulus *E*_MIN_ and *E*_MAX_, which confirmed model previsions as tensile load increments up to 100% and toughness increments up to 69%. Comparing the results from [25] and [26] it can be observed how symmetrical elastic modulus bond layer tailoring is more effective than monotonic tailoring. This is true even for geometries that have non-symmetrical stress distributions under tensile loading due to the geometrical configuration of the joint.

**Figure 5:**
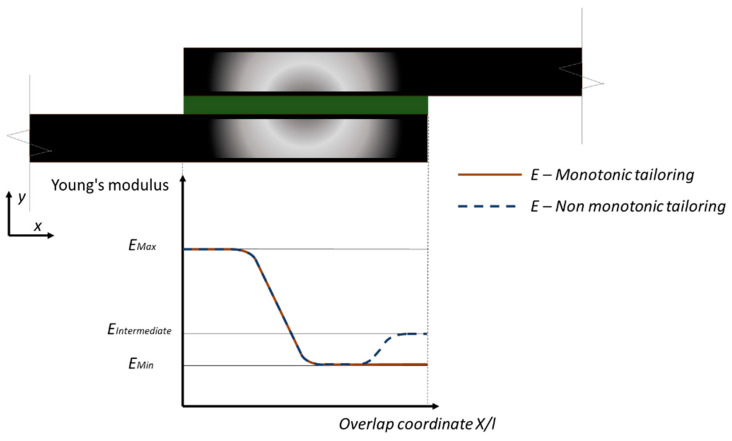
Adherend Young’s modulus tailoring using digital materials SLJs [30].

Ubaid et al. [30] focused on the use of stiffness tailoring of the adherend to affect the stress distribution in the bond line and improve the overall joint carrying capacity. Authors took advantage of the DMs to control the material properties of the adherends. The 2D FEM models were implemented in order to investigate different tailoring configurations in the x and y directions for the SLJ geometry, as shown in Figure 5. The results of 1D tailoring showed that this design strategy led to an increase of secondary bending under load with consequent peel stress increases. With 2D tailoring it was possible to optimize the material stiffness and improve the stress distribution and minimize the bending. The best configuration was found to be the one with non-monotonic tailoring as shown in Figure 5, with a reduction of peak peel stress by 57% and an almost constant shear stress distribution. Experimental testing on standard and tailored SLJs demonstrated the effectiveness of this configuration, with an improvement of 13% of the failure load, 20% of displacement at failure and 50% of the joint toughness confirming how 2D elastic modulus tailoring can be used to control the bending in the joint.

#### 2.1.2. Locally Controlled Properties

This subsection presents works that use, as design strategies, the introduction of tailored porosities, macro voids or cavities in the adherends to locally modify their mechanical properties. This allows us, for example, to locally vary the material density [31] and thus modify the overall joint response under loading. These design approaches can be considered an extension of the concept of varied densification from functionally graded additive manufacturing (FGAM) [32], in which the voxel oriented material modelling shifts the design focus from the geometrical dimensions to through-the-thickness structures in order to maximize the performance of the components.

**Figure 6:**
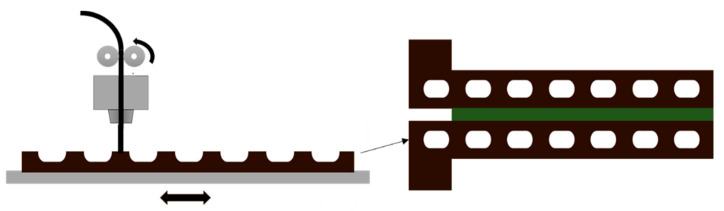
Adherend bio-inspired stiffness tailoring using a sub-surface geometry as proposed in [33,34,35].

A first feasibility study on crack trapping using AM for bonded joints was made by Alfano et al. [33]. In this work, the authors used simplified 2D sub-surface structures (Figure 6) bio-inspired by the Balanus Amphitrite in order to obtain a toughening effect. The aim was to improve the reliability and the damage tolerance of AM layered materials as these are relevant factors for use with AM components in structural applications. The authors used the powder bed fusion process and polyamide (PA) to manufacture adherends with square and circular cross section structures through-the-thickness. Double cantilever beam (DCB) specimens were produced by bonding the resulting adherends with the Hysol 9466 epoxy adhesive (Henkel, Düsseldorf, Germany) without any surface modification. Experimental testing to assess the effect of the sub-surface channels on the energy dissipated by the joint were carried out. A global increment in load-displacement values were registered for the DCBs tailored with channels with respect to standard DCB adherends. The magnitude of the increment was distinct for the different investigated shapes using the digital image correlation (DIC) technique.

These first promising results led to further investigation carried out by Morano et al. [34]. At first, FEM models for three different channel shapes were proposed and validated with experimental data in order to correlate the sub-surface geometry with the crack propagation behavior under mode I loading. DCB specimens, in the same configuration proposed by Alfano et al. [33], were fabricated and tested. Using FEM simulation and the virtual crack closure technique (VCCT), the strain energy release rate was evaluated for both standard and tailored configurations. It was observed that the energy release rate was affected by the crack position with respect to the material distribution of the sub-surface geometry. By comparing the results obtained with the different channel shapes, it was established how channel shapes have a relevant effect on crack propagation and, therefore, a parametric study on sub-surface geometry was carried out in a second work [35]. It was found that increasing channel height, width and pitch has a positive effect on joint performance. Larger values for these geometrical parameters, lowering the adherend stiffness, led to the nucleation of secondary cracks that significantly increase the dissipated energy. However, these values are limited due to buckling phenomena and consequently there is an adherend strength reduction. The conflict between strength and toughness is a well-known engineering problem [36], but recent works attempted to address it by developing new materials or material deposition micro bio-inspired patterns [37].

The work by Afferrante et al. [38] can be considered as complementary to the works by Alfano et al. [33] and Morano et al. [35] as it investigates the effect of 2D sub-surface structure with loading in different directions with respect to the tailored structures. The authors studied how it is possible to control the joint strength using the crack trapping mechanism and controlling the adherends’ stiffness. In a direction orthogonal to the embedded channel structures, the toughening effect is confirmed. On the other hand, crack propagation parallel to the channels is affected in a detrimental way. The direction-dependent adhesion properties must be taken in account while designing the AM tailored joints in order to avoid failure under accidental loading of the structure, but they also can be exploited to enable new applications, for example in robotic manipulation systems.

#### 2.1.3. Adhesive Tailoring

AM enables control over the material at voxel resolution and this, if coupled to MMAM, can be used to create joints with tailored physical and mechanical adhesive properties. One of the main engineering challenges in tailoring adhesives is to obtain a seamless variation in the material properties in order to obtain a functionally graded adhesive (FGA) behavior [19]. FGA can be obtained by a continuous change in material composition and/or the microstructure along the overlap of the joint. The practical methods to obtain FGA adhesives are to mix adhesives with particles of different properties in different volumetric percentages, to create a non-uniform reinforcement distribution in the bond line or to apply different localized curing cycles. Usually the aim is to optimize the stress distribution in the bondline, lowering peel stresses at the overlap ends.

Redmann et al. [39], using AM based on photopolymerization, developed a joining method, similar to the AddJoining one [12], for composite reinforced fiber polymeric adherends. The authors assessed the feasibility of AM adhesive deposition using the b-stage curing epoxy EPX81 (Carbon Inc., Redwood City, CA, USA) and adherends made of NB-EP4030-D (Mitsubishi Chemical Holdings Corporation, Tokyo, Japan) pre-impregnated woven carbon fiber. The adhesive material was deposited in a controlled way on the adherends using the AM process based on the continuous liquid interface production (CLIP). After deposition, the adhesive was partially cured with ultraviolet (UV) light. Afterwards the substrates were assembled to form a SLJ and the cure was finished using a thermal cycle. Reference values were provided by testing monolithic SLJs of NB-EP4030-D carbon fiber reinforced polymer and adhesively bonded SLJs with 3M DP190 (3M Company, Maplewood, MN, USA), fabricated with the same dimensions. Micro-computed tomography (micro-CT) testing assessed how AM joints had a nominally void free adhesive layer in respect to manually deposited adhesive. Mechanical testing did not point out any relevant differences in terms of maximum shear stress, while lower strain at failure and lower toughness was identified for the manually assembled joints. Experimental testing proved that it is possible to deposit the adhesive in a controlled way using AM but the use of a b-stage curing adhesive places limitations on adherend-adhesive compatibility and on the processing temperatures.

The work of Dahmen et al. [40] was based on the work by Redmann et al. [39] and used the same 3D printing set up and materials to assess if the proposed AM adhesive deposition method could be suitable for industrial application. As a case study, the authors considered a T-joint configuration, a non-standardized joint but highly relevant in aerospace industry applications, as it is commonly used in the spar-skin joints of aircraft wings.

**Figure 7:**
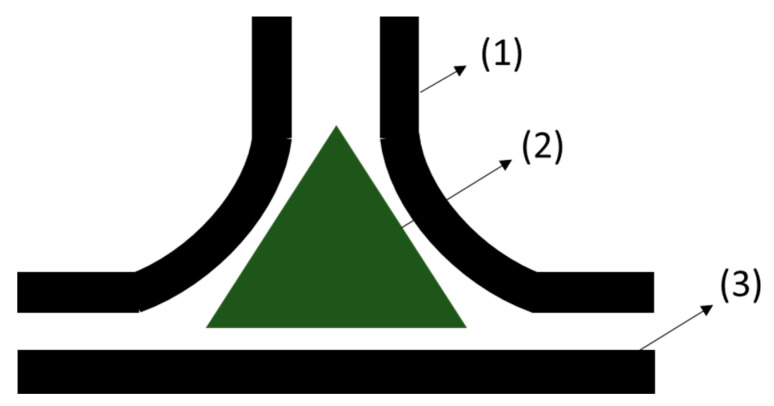
2D view of the components of a T-joint assembly, webs (1), deltoid (2) and platform (3).

The T-joint solution (Figure 7) is designed to transfer out of plane loads and is composed of a base platform, a bent vertical structure with a stiffening function integrated in the shape and a deltoid, which is a filler to stabilize the webs. It was assessed that the deltoid material and shape are a relevant factor to improve the joint load carrying capacity, but the actual manufacturing process, based on the manual pre shaping of the deltoid material, cannot guarantee reproducibility or a void free joint. The use of an AM process to introduce partially cured adhesive was identified as a possible solution to address the limitations of the manual process and to obtain highly optimized joint shapes.

**Figure 8:**
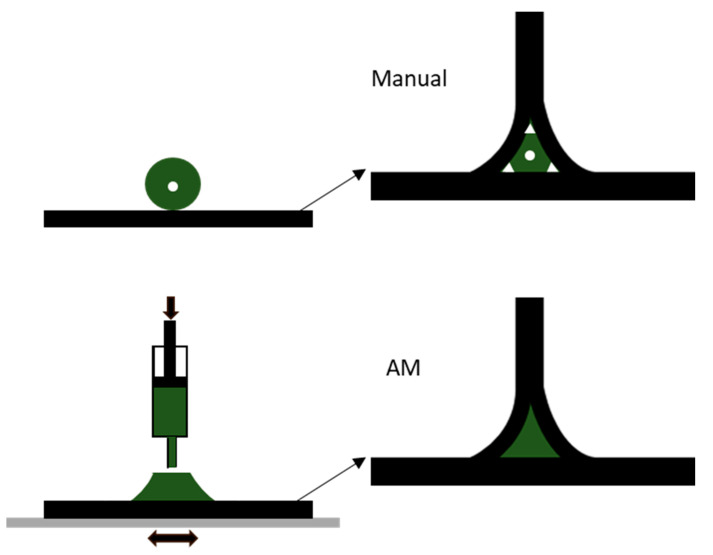
Manually pre-shaped deltoid vs. an AM processed pre shaped deltoid. AM T-joints have better geometrical accuracy and less porosity.

The joining process was performed using three different processes to manufacture the deltoid: adhesive bonding with AM, a manual process with adhesive bonding and a manual process with pre impregnated carbon fiber (Figure 8). At first, SLJs were manufactured in order to tune the process parameters and to set a reference using a standardized testing method. Results confirmed those reported in [39]. Failure modes were mixed, exhibiting both adhesive and cohesive failure of the adhesive for the two bonding-based solutions while delamination occurred in the co-cured pre impregnated joint. Delamination was restricted to the bondline zone and did not progress to the adherends. An initial visual inspection confirmed the superior quality of the AM joints, without the porosity and voids associated with the manual adhesive deposition or the rolling process of the pre impregnated fibers (Figure 8). Manually processed T-joints, tested under a pull-out load, failed at 284 N, while using AM to apply adhesive the load at failure increased by 34%. The use of pre impregnated fibers was quite effective, showing a 47% increment over the manual reference. The authors pointed out how, considering relevant factors such as the different base material mechanical properties, joint defects and result reproducibility, the AM process appears to be the best solution, especially since the curing processes were not the subject of an optimization process. Moreover, the geometrical accuracy of the AM process could be used to tailor bonded joints for several specific applications, for example, in joining components with optimized and complex overlap geometries or hollow structures.

The work by Niese et al. [41] assessed the feasibility of using powder bed fusion to tailor the adhesives in both deposition patterns and in its physical properties, such as electrical conductivity. However, the authors warned of high porosity in the materials due to the process.

The work by Schmidt et al. [42] completed the previous works [39,40,41] by assessing the effect of various deposition strategies on the physical and mechanical properties of UV and thermally cured adhesives. Schidt et al., changing the AM process, were able to overcome the limitation of SLA and manufacture flawless bond lines, confirming the results obtained in [39]. The aim of the work is the same presented by Niese et al., which is to create a single process for the manufacture of complex mechanical, electrical and optical conductive systems using AM and direct printing. The authors designed a custom-made set up in order to evaluate the effect of build direction, the edge along which the layered adhesive stacking is performed, the raster angle and the deposition pattern of the material in the single layer, on the physical and mechanical properties of adhesives processed via 3D printing. The investigated adhesives were five dual curing, four commercial solutions, epoxy/acrylate Loctite 3217, acrylate Loctite Eccobond UV 9052 (Loctite Corporation, Düsseldorf, Germany), polycarbaminoacid Delo Dualbond AD346 (Delo Industrie Klebstoffe GmbH & Co. KGaA, Windach, Germany), and one experimental epoxy adhesive. All adhesives contained particles that provide them with thermal and electrical conductivity. Reference values were obtained for the mechanical properties using specimens fabricated in a jig and using a single cycle cure adhesive, the Dellomonopox GE7985 epoxy (Delo Industrie Klebstoffe GmbH & Co. KGaA, Windach, Germany). Analysis on the experimental results showed that the different raster orientations do not have a significant effect while the build direction does, leading to strength increases of up to 70%. Observing the fracture surfaces allows us to understand how a longer UV pre curing period enhances the layering effect, anisotropy and particle deposition, with a positive effect on thermal and electrical conductivity. It is worth noting that the rheological behavior of investigated adhesives was shear thinning, which means no flow at rest, and that UV pre curing enables high geometrical details without support and non-dependent build direction. These characteristics are relevant for manufacturing components with complex geometries.

**Figure 9:**
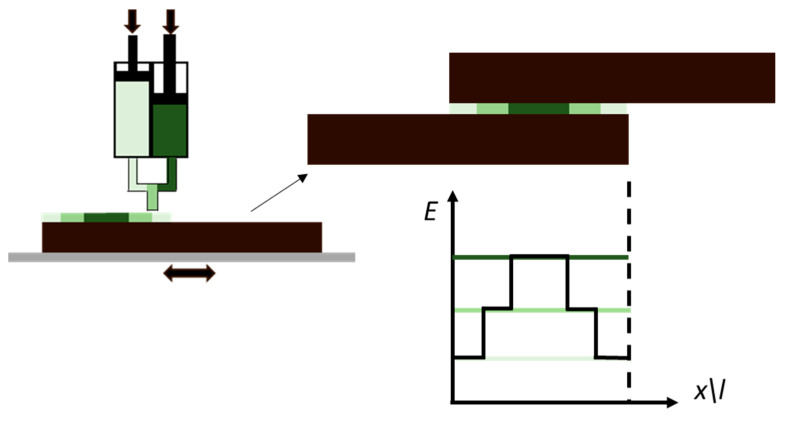
Adhesive modulus tailoring using a stepwise design strategies and second generation acrylic adhesives [43].

Sekiguchi et al. [43] applied a strategy that combines the adhesive deposition process proposed by Redmann et al. [39] and the work of Kuman et al. [24] for adhesive modulus tailoring along the bondline (Figure 9). These techniques were applied to manufacture SLJs that were later tested under static and low-cycle loading. The authors took advantage of a characteristic of the second-generation acrylic adhesives, where varying the mixing ratios of the two base agents can lead to different mechanical properties. Using agents with different mechanical properties after curing, the stiff G672-ISP and the flexible LDC-141 (Denka company, Tokyo, Japan), in four different ratios 4:6, 5:5, 6:4 and 7:3, the authors were able to tailor the adhesive bulk properties, from 3.72 MPa, 9.06%, 13.8 MPa to 13.7 MPa, 2.81%, 19.5 MPa respectively for elastic modulus, stress at yield point and strain at yield point.

To control the mixing ratio of agents during the adhesive deposition at the overlap, a special apparatus was purposely developed [44], making use of a concept already in use for 3D printing of clay and concrete [45], where the extruder is replaced by an adduction system that allows us to extrude fluids for the liquid deposition modelling process (LDM). Using this apparatus, flexible adhesives were extruded at the end of the overlap end while a stiff adhesive was extruded at the overlap center. This is achieved by increasing the volumetric content of the stiff agent every 5 mm along the overlap length, creating a SLJ with tailored adhesive properties. Reference SLJs were manufactured with the same geometry and non-tailored adhesives using the two extreme values of the mixing ratio (the stiffest and the most flexible). Specimens with the graded adhesive outperformed the flexible and stiff non tailored adhesive joints in quasi static testing, with an increase in failure load of 16.3% and 21.4%, respectively.

While tailoring the adhesive properties along the overlap was already proven to be an effective solution to improve the performance of bonded joints [19], AM processes could be extended to other aspects of a bonded joint to further improve performance. This can be achieved with a finer control over the final geometries, adopting voxel-oriented material tailoring, enhancing reproducibility and lowering the porosity in the adhesive. The next main challenges will be in engineering the proposed methods for different adhesives and different scales, from large aircraft wings [40], integrating the deposition of the long fiber reinforced matrix, to micro scale, electronic applications using new tailoring approaches such as the one presented in [46] where a patterned adhesive, controlled by droplets in size from 0.5 to 6.0 µL, outperformed a continuous adhesive with an increment of ~70% in shear strength.

### 2.2. Overlap Geometry Tailoring

This section presents and summarizes works which aim to improve the performance of adhesive bonded joints performance with an optimized geometrical design of the adherend in the overlap area. The approaches presented here were often originally proposed for laminate materials [20,47], which have many similarities to AM processed components, such as a layering effect and an anisotropic behavior under load [48].

**Figure 10:**
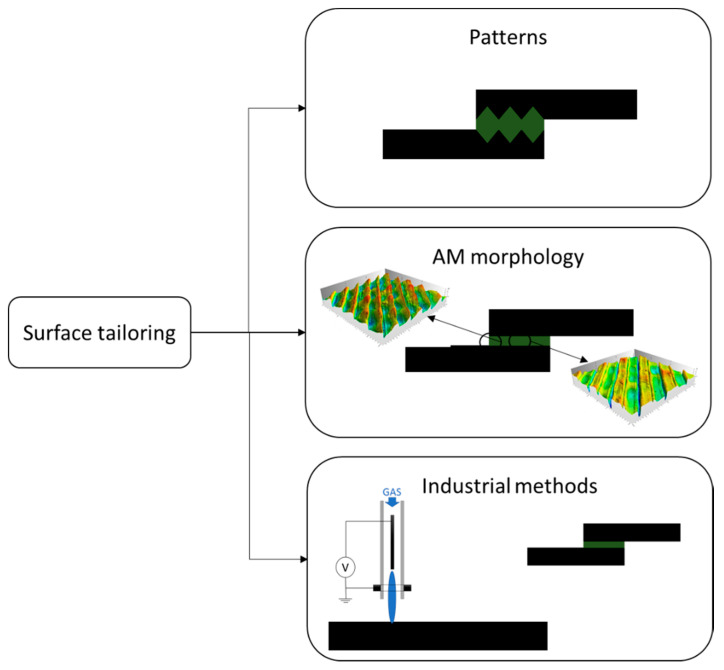
AM design strategies for tailoring the adherend at surface overlap.

The AM design strategies to increase the bonding area or to modify surface properties, presented in this section and those that follow it, are shown in Figure 10. At macro-scale, adherend modifications can be achieved by including features such as pins or wavy interfaces. At micro-scale, AM process parameters, such as layer height or nozzle temperatures, can be used to finely modify surface properties, leading to changes in the morphology. Some surface modifications suitable for industrial contexts were also investigated.

**Figure 11:**
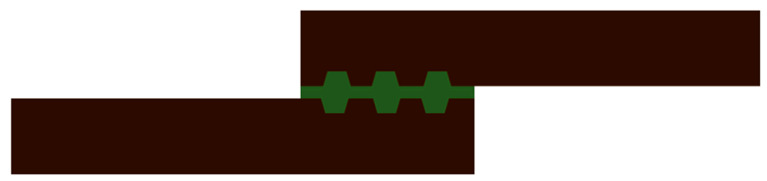
AM adherends with enhanced bonding area in the adherend volume [49].

Spaggiari et al. [49] explored the effect of a sawtooth patterns applied at the overlap area, generating the geometry while manufacturing specimens with fused deposition modelling (FDM) Stratasys process (Figure 11). Acrylonitrile butadiene styrene (ABS) was used for the specimens as well as a variety of structural epoxy adhesives with slightly different elastic moduli. The authors investigated the effect of zig-zag patterns with different orientations, (0°, 45°, 90°) with respect to the loading direction and compared the output with a monolithic reference SLJ. Data analysis revealed that the adhesive stiffness is the only relevant effect in the investigated configuration, as the joints assembled with ductile adhesive outperformed all others. Furthermore, it is worth noting how ABS polymeric adherend and epoxy resin bonding does not require additional surface modifications besides solvent cleaning. Failures were always cohesive in the adhesive or in the adherend. Thus, the sawtooth morphology is to be discarded as its shape does not provide mechanical interlocking and is in fact a stress concentration feature. However, it still has some practical potential as a shape optimization for this kind of AM patterns at overlap has yet to be published in the literature.

Cavalcanti et al. [50] expanded upon the work of Spaggiari et al. [49] by investigating the effect of sinusoidal tailoring of the overlap coupled with different building directions. The authors, using fused filament fabrication (FFF), built polylactic acid (PLA) adherends in two different directions, flat wise and edge wise, and manufactured SLJs using an epoxy adhesive. A good degree of compatibility between the PLA polymer and the epoxy resin was observed, as all joints had failure in the adherends. Additional remarks were drawn by observing the failure surfaces. Specimens built in the flat wise direction or with a standard overlap geometry presented delamination failure, while the specimens built in the edge wise direction or with a sinusoidal overlap presented failure through the thickness, illustrating how component positioning can strongly affect the mechanical properties of layered constructions [51].

Garcia-Guzman et al. [52] investigated the effect of the structured interfaces seen in the previous works by Spaggiari et al. and Cavalcanti et al. [49,50] using DCB testing. This experiment was carried out to determine how morphological patterns on the surface of the adherends influence the fracture toughness. The authors parametrized a recurrent trapezoidal pattern as a function of discrete values of amplitude, wavelength and aspect ratio. Tests carried out on PA glass fiber reinforced adherend, manufactured with a MarkOne (Markforged, Watertown, MA, USA) 3D printer and bonded with structural adhesive, led to an increase of the critical energy release rate from 100% up to 800%, when compared to the flat DCB configuration. An analytical model was developed to understand the physics of the fracture process and to support the selection of the optimal pattern configuration. It was observed that the tooth morphology increases the effective bonding area and modifies the direction of the crack propagation as a function of the tooth slope. Under the hypothesis of constant strain rate, it was possible to distinguish between the energy dissipated at the flat part of the trapezoidal tooth and the energy dissipated at the slope and to use a failure criterion derived from the Benzeggah-Kenane one [53]. Correlating experimental data to the components of the dissipated energy showed that a higher amplitude to wavelength ratio corresponds to higher fracture toughness. This is related to a change of the fracture from pure Mode I to mixed mode which forces crack propagation to travel longer distances along the vertical and horizontal directions and thus absorbs more energy.

**Figure 12:**
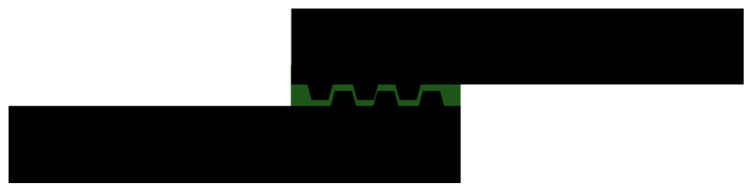
Adherends with a AM deposed pattern at the overlap in the bondline volume [54].

Garcia et al. [54] investigated the effect of an AM tailored macro pattern on an adherend that protrudes into the bonding line (Figure 12). The authors used the FDM process to create a tailored texture on carbon fiber-reinforced polymeric (CFRP) adherends with the AddJoining method [12]. This approach has the advantage of being compatible with a wide range of materials and works with any adherend, independently of its manufacturing process. The effect of different spacing of ABS reinforcements, having uniform trapezoidal cross sections along the width and height similar to the adhesive layer thickness, was numerically modelled and experimentally validated. The numerical analysis pointed out how the reinforcements modify the stress distribution. Experiments quantified the positive effect of the macro pattern on joint performance, changing the failure mode from adhesive to cohesive and attaining a minimum performance improvement of 43%. For every reinforced configuration studied, an increment in strain was found.

Bürenhaus et al. [9] investigated different tailored overlap configurations, such as the scarf joint, the finger joint and the tongue-groove joint against more standard joint geometries such as the SLJ, butt joint and T-peel joint. Specimens were manufactured and numerically modelled, using a composite-like material approach presented in [48]. The authors compared the results of these approaches to the ones obtained with a reinforcement pattern on the surface designed as presented in [54] by Garcia et al. Adherends were manufactured in the various geometries using FDM with polyetherimide (PEI) material. The adhesive used (Loctite LM-21 HP) had excellent compatibility with PEI without surface treatment. Preliminary screening with SLJs showed cohesive failure in the adhesive. The base geometry was designed according to DIN-EN-ISO 1465 [55] with a thickness of 1.778 mm, width of 25 mm and an overlap of 12.5 mm. Experimental testing confirmed the dominant effect of changes in the geometrical configuration on the texture obtained by the manufacturing process. The maximum joint strength of 35.45 MPa was achieved with the butt joint configuration, while T-peel had the lowest joint strength value with about 13 MPa. Among the joints with tailored overlap geometry, the scarf joint had the best performance improvement of about 66%, despite an almost unchanged overlap area. In the specimens manufactured according to the work of Garcia et al. [54], adherend failures occurred at low loading due to stress peaks at the pattern locations.

Data on the effects of overlap tailoring provided by the previously described works have been completed by the investigations carried out by Fieger et al. [56] on tailoring using 3D and 2D pattern geometries with constant cross section along the adherend width. Four different patterns were designed to address the low adhesion of adhesives to PA. The design takes in account different overlap tailoring strategies, namely maximizing bonding area using coupled protruding-hollow dimple-shapes, modifying the bondline with 2D structures equally spaced along the overlap, seeking mechanical interlocking of adhesives in the adherends using undercut or between adherends with snap fastener like structures. The SLJ results, in agreement with the ones from Spaggiari et al. [49], indicated a beneficial effect of increasing the bonding area. The tensile shear strength increased by 44% with respect to flat-standard SLJ. The 2D structure in the bondline was found to be unsuitable. This result is in apparent disagreement with the findings of Garcia et al. [54] but could be explained by the different and uniform spacing of the AM structures in the bond line. Finally, the maximum improvement in tensile shear strength is achieved with the configuration that combines adhesive bonding and mechanical interlocking between the adherends, indicating that this design strategy should be the target of further research.

The use of mechanical interlocking in AM bonded joints is still an open topic of research and few works are available on this specific subject, even if it seems to have a mostly positive effect on joint performance. This is probably due to the limitations on buildable geometries and the geometrical tolerances achievable with current AM processes. However, recent studies on the effect of the positioning and AM process developments are now enabling these novel design strategies. An example of mechanical interlocking applied to AM bonded components can be found in the work of Jilich et al. and Frascio et al. [57,58], where pins were used for adherend alignment, adhesive thickness control and to create a snap-like connection. Another relevant work on the design of AM snap connection for AM can be found, although without the use of adhesive bonding. Rossing et al. [59] addressed the low compatibility between silicones and other materials with mechanical interlocking and MMAM, devising a solution that dispensed the use of primers. Possible future works could focus on assessing the feasibility and the effectiveness of adopting AM joints solutions previously investigated for other processes and materials, such as pinning, tapering or filleting with integrated design in the AM tailored adherends.

### 2.3. Overlap Surface Modifications

This section reports works that focus on surface modifications in AM joints, carried out in order to obtain performance improvements. According to Packham [21], adhesion mechanisms are described by three main supporting theories; the absorption theory that focuses on the correlation between wettability and covalent and van der Waals forces, the mechanical theory that focuses on the mechanical interlocking that occurs when the adhesive penetrates in the adherend morphology and, lastly, the diffusion theory that is based on polymer chain dynamics and the compatibility between the different polymers. It is worth noting that, at time of writing, recent literature [60] is questioning the chemical effect of surface modifications on bonded joints performance, pointing out that performance variations could be only correlated to the morphology modifications at nanoscale.

AM offers the possibility to implement integrated surface modifications. This can be done, for example, by mounting an atmospheric plasma torch on the nozzle or to affect the surface properties through the printing set up.

**Figure 13:**
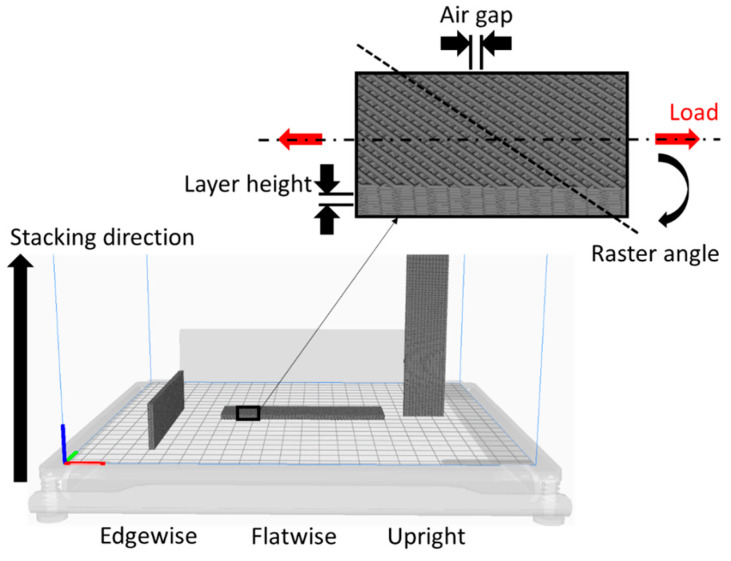
Overlap modifications through the parameters of build set up, investigated in [9,50,61,71,72,79].

An AM integrated approach was first presented in the work of E. Dugbenoo et al. [61], where the authors increased the effective bonding area by using the air gap AM process parameter (Figure 13).

By setting a partial infill at the overlap interface, while compiling the G-code programming language, it is possible to obtain optimal surface morphology avoiding post processing. Thus, it is clear how this approach could be advantageous for a reduction of production costs. By setting an offset of ~120 µm in the material deposition pattern, the authors obtained a 50% infill density at the adherend–adhesive interface that led to the increase of the bonding surface area by ~150% and enhanced the interlocking effect. To assess the effect of this design on actual joints AM SLJs were manufactured with printed adherends composed of long fiber, carbon, Kevlar and glass, reinforced PA matrix supplied by Markforged. SLJs were manufactured using a two-component epoxy adhesive (EM9500 and EM9520, Kumho P&B Chemicals Inc., Seoul, South Korea) to join mechanically abraded and air gap tailored adherends. Experimental data identified a joint strength increase of up to ~145% and a joint toughness increase of up to ~820% for tailored joints, values which are dependent on the nature of the material being used as reinforcement.

Fieger et al. [56] experimentally evaluated the effect of four different surface treatments on powder bed fusion processed PA adherends. Adherends were treated with flame treatment, corona treatment, atmospheric plasma treatment and chemical etching treatments and then used to manufacture bonded joints with four different adhesives. SLJ testing highlighted changes in the failure mode from adhesive to cohesive and an improvement in tensile shear strength. Maximum tensile shear strength was measured for flame treated SLJs with an improvement of over 80% with respect to the reference. It must be noted that the results have large dispersion, suggesting low process reproducibility.

Leicht et al. [62] investigated the effects of the construction direction (Figure 13) and surface modifications on the tensile strength of adhesive bonded joints made of powder bed fusion processed PA adherends and epoxy resin. Adherends were fabricated with a geometry compliant to the Centrifugal Adhesion Testing Technology (CATT) procedure described in [63], that uses a centrifugal force to apply the load and satisfy DIN-EN-ISO 15870 standard [64]. Three different epoxy adhesives with different mechanical properties were investigated, DELO-DUOPOX SJ8665 (DELO Industrieklebstoffe GmbH & Co. KGaA, Windach, Germany), 1277 (Sika Schweiz AG, Zurich, Switzerland) and Easy-Mix HT 180 (WEICON GmbH & Co. KG, Muenster, Germany). The joints were fabricated with adherends subjected to chemical smoothing, atmospheric pressure plasma (APP) and the combination of the two treatments. Untreated substrates were also used as a reference. Experimental testing showed that both adhesives had a similar response to the surface modifications under tensile loading, with a decrease in tensile strength after chemical smoothing, but increase after APP and after the combination of the two treatments. To understand why chemical smoothing has a negative effect on the load-carrying capacity of the joints, the authors performed CT scans on the specimens. This analysis demonstrated how chemical smoothing erases the porous morphology resulting from the melted powder particles, preventing mechanical interlocking between the adhesive and adherend A maximum tensile strength variation of 300% was found with respect to the non-treated configuration.

Bürenhaus et al. [9] investigated the effect of different surface modifications using industrial treatments and the build parameters of the AM processed (Figure 13) PEI adherends. The tested industrial modifications were ethanol cleaning, abrasion using P80 sandpaper, blasting with glass beads and APP. The raster orientation was selected in order to assess the effect of the directionality of the AM surface texture with respect to the loading direction, 0° parallel to load direction, 45° baseline orientation and 90° orthogonal to the load direction. WELDYX Polyplast acrylate resin (GLUETEC Industrieklebstoffe GmbH & Co., Greußenheim, Germany) and DELO-PUR 9694 polyurethane were selected for this study because they generated adhesive failures without treatment. Experimental data demonstrated that ethanol cleaning and mechanical abrasion are ineffective, while APP and blasting provide similar results with SLJ shear strength improvement of about 110% with respect to the untreated configuration. Surface modifications created using the AM build parameters have the advantage of being directly integrated in the manufacturing process, but they negatively influence the mechanical properties. The authors tailored only the top layers in order to minimize possible interaction effects, but delamination occurred when increasing the airgap to 0.5 mm. The authors pointed out that the use of the scarf joint, coupled to the design freedom of AM, allows one to easily achieve overlap tailoring in these geometries, leading to excellent performance with regard to the maximum transferable forces and reaching utilization factors of over 80%.

Li et al. [65] investigated the effects of mechanical abrasion and APP on adherends made of high performance AM processed polymers (PEI, polyether ether ketone PEEK) and carbon fiber reinforced PEEK using two different adhesive technologies, a film solution by 3M (AF163-2) and a liquid solution by Henkel (Ea9380.05). The adherends were manufactured using an FFF printer with a geometry according to GBT 33334 standard [66]. SEM and confocal laser scanning microscopy (CLSM) assessed how the typical stripe-like morphology conferred by FFF was erased by the sandblasting treatment, leading to uniform value of surface roughness (*Ra*) while the use of APP led to almost no changes. SLJs were fabricated and tested according to ASTM D5868 [67] using solvent cleaned, sandblasting and APP treated adherends. Data from mechanical testing demonstrated the necessity of a proper surface treatment, as the failure surfaces of solvent cleaned substrates were always adhesive in nature. The PEI adherend reached the maximum shear load after sanding and APP, with mixed adhesive cohesive failures, while PEEK and carbon fiber-reinforced PEEK reached the maximum shear load after APP, with increments up to +400%.

Yap et al. [68] investigated the effect of curing the assembled AM SLJs using a thermal cycle. The application of heat can be beneficial, accelerating adhesive cure, and improving mechanical performance. It enhances the phenomena of crosslinking in the adhesive and of diffusion between the layers of the AM adherends. Materials selected to build the adherends were two different engineering polymers, the UV resistant acrylonitrile styrene acrylate (ASA) and the CFRP PA. Two adhesives suitable for bonding polymers were selected, the slo-zap cyanoacrylate by ZAP (Ac Marca, Barcelona, Spain) and E-20 HP epoxy by Loctite. Adherends and monolithic SLJs, used to set reference values, were manufactured in compliance with ASTM D3163-01 [69] and ASTM D1002-10 [70] using FDM. The adherends were prepared with two different surface preparations, solvent cleaning and mechanical abrasion, while the SLJs were cured at room temperatures and at 80 °C. For both materials, the maximum tensile load was achieved in the monolithic configuration. SLJs bonded with the cyanoacrylate adhesive always showed cohesive failures, maximum strength of bonded SLJs was obtained for the solvent cleaned surface with 1.7 and 2.7 kN, respectively for ASA and PA, because the heat cycle led to adhesive degradation. On the other hand, SLJs bonded with the epoxy adhesive always failed adhesively, indicating an inadequate surface preparation for structural applications. Indeed, after heat treatment the PA SLJs reached a maximum shear load of 1.7 kN, 2.5 times lower than monolithic reference value.

Kovan et al. [71,72] used FFF to manufacture SLJs and butt-joints with PLA substrates to experimentally assess the effect of changing printing orientation and layer height (Figure 13). The authors design strategy is based on the observation that AM parameters affect the component quality, and in particular the surface morphology [73,74,75]. This can be used to enhance the mechanical effect of adhesion [21,76]. Adherends were designed according to ASTM D3165 [77] for the SLJ geometry and according to ASTM D2094 [78] for the butt joint geometry in three different layer stacking directions, upright, edgewise, flatwise and with three different layer heights, 125, 250 and 500 µm. Preliminary mechanical characterization showed how the stacking directions of the layers affected the material modulus though diffusion quality and roughness orientation. Each of the joint configurations had a distinct optimal setup to maximize the load carrying capacity. The maximum shear load for SLJs was achieved for the edgewise direction and 250 µm layer height with a performance improvement of 200% with respect to the worst configuration (edgewise direction and 500 µm layer height). For the butt joint, the maximum load value was obtained for the minimum layer height with an improvement of 640% with respect to the lowest load value measured using adherends with maximum layer height. The authors pointed out that build time and maximum joint load are non-linearly correlated to the levels of the investigated factors.

Kariz et al. [79] investigated the effect of the 3D printing set up on the adherends’ morphology for heterogeneous polymer-wood joining. Using FFF equipment and ABS filament, the authors created SLJ adherends, designed according to EN 205:2003 [80], with three different layer heights, 90, 190 and 390 µm. A reference was set using the same 3D printed adherends and erasing the surface morphology by acetone vapor chemical smoothing. SLJs were assembled without further surface modifications using three different adhesives, Mitopur E45 PU1K (PU 1K; Mitol d.d., Sezana, Slovenia) and Bison Power (PU 2K; Bison International, Goes, Netherlands) polyurethane adhesives, and DORUS KS 217 hot melt adhesive (HM; Henkel, Düsseldorf, Germany). Analyzing the shear strength of the different SLJ configurations some remarks can be drawn. The Bison Power adhesive had the highest shear strength in each configuration while Mitopur E45 had the lowest. Acetone smoothed specimens had the lowest overall shear strength, showing how the AM derived morphology has a positive effect.

It is worth noting that vacuum-ultraviolet (V-UV) surface treatment at date of writing was not investigated for AM adherends.

## 3. Conclusions

Recent developments in additive manufacturing processes and in innovative materials have expanded the use of additively manufactured components to many new engineering fields, leading to an increased interest in the use of this technology. In particular, the joining of additively manufactured components has been a key area of study, as this technology is essential for manufacturing structures of increased complexity.

Different design strategies have been investigated to take advantage of the beneficial effect of voxel size control provided by additive manufacturing on the geometry and material properties.

Most of works focused on additively manufactured adherend tailoring. Kumar et al. [23,24] and Khan et al. [25,26,27] in their works modelled the stress distributions of joints using functionally graded materials achieved with digital materials and Ubaid et al. [30] experimentally assessed load carrying and toughness improvements due to a modified strain field distribution in the bondline using single lap joints and digital image correlation.

Other authors focused on the so called geometrical-complexity-for-free provided by additive manufacturing processes and used tailoring of adherends at the bonding interface and in the thickness. The first approach is used to create a desired pattern able to provide mechanical interlocking and increase the area at the interface between adherend and adhesive. Several authors [49,50,52,61] used geometrical patterns indented in the adherend thickness to maximize the overlap area, while others [23,54] used patterns protruded in the bondline to seek mechanical interlocking and to alter the strain distribution in the adhesive.

The second approach draws inspiration from organisms which show high adhesion and toughness. Using a fracture mechanics-based approach, several authors [33,34,35,37] developed through the thickness structures able to delay crack propagation and the failure of the joints.

The results of these works provided the first quantification of the effects of tailored additively manufactured adherends and highlighted the advantages and disadvantages of this design approach. Major limitations are due to the minimum size of the printable features, inherent to a given additive manufacturing process specifications, and the effect of the tailoring on the adherend stiffness. Indeed, an unsuccessful tailoring procedure can increase the susceptibility to bending, leading to increased peel stress in the bondline and causing failure at loads lower than that of a non-tailored joint configuration.

New developments in additive manufacturing processes, such as the multi material additive manufacturing and the use of new materials, are enabling different design solutions, such as the creation of functionally graded adherends or embedded sensors, although they urgently require the development of new testing methods and standards to address the resultant complex material response. Available results indicate that this approach can improve joints’ performance, although it is still common for joints manufactured using additive manufacturing technology to fail predominantly by adhesive failure, pointing out that further performance improvements can be achieved by improving the quality of surface treatments and material compatibility.

A possible solution for these limitations could be the combination of several different design approaches for the manufacture of the adherends or the use of surface modifications. Some of the studies applied surface modifications which are already in industrial use [56,62,65,68], while others [9,50,61,71,72,79] explored how the printing parameters affect the physical and the mechanical properties of the polymeric adherends. Plasma treatment was assessed as the most effective surface modification for this purpose as it is suitable for use in hollow structures and has the potential of being process integrated. For example, an FFF printer can include a plasma torch on the nozzle. On the other hand, while tailoring the printing parameters does not require any additional equipment or post processing, it can be a highly complex process, as the interaction between different parameters can lead to unexpected and undesirable results.

Some authors explored the feasibility of using additive manufacturing to tailor the bondline by controlling the adhesive composition or shape [39,40,41,42,43]. The main benefits of this approach are the compatibility with non-additively manufactured adherends and the improved joint quality, as the adhesive layer is relatively free from porosities and possesses high geometrical accuracy and reproducibility. Such local control has the potential to enable the manufacturing of true functionally graded adhesives by controlling adhesives’ composition, adhesives’ mixing, or the dispersion of reinforcements in the adhesives. This concept has been successfully demonstrated in laboratorial settings, but no commercial solution is available at the time of writing this document.

Finally, tailoring additively manufactured adherends and adhesives, following a design for additive manufacturing approach, has proven to be an extremely useful, yet relatively unexplored, solution to improve the performance of adhesively bonded joint. While many researchers have laid the foundations for more extensive use of additive manufacturing in conjunction with adhesive bonding, additional research is still required to enhance manufacturing reliability and repeatability. Another important conclusion of this work is that aspects related to the degradation and long-term durability of polymeric additively manufactured bonded joints are relatively unstudied. This represents a topic of great interest for industrial applications and must certainly be an important subject of research in the coming years.
